# The Upregulation of NDUFB3 Is Implicated in Mitochondrial Dysfunction and Neuronal Apoptosis in Ischemic Stroke

**DOI:** 10.3390/cells15121071

**Published:** 2026-06-12

**Authors:** Shuyue Cheng, Zeyue Mu, Feng Zhang, Jianyou Song, Jiapeng Shao, Yunqi Yan, Anastasios A. Daskalakis, Yunjie Wang, Bin Zhang, Yashuang Jiang, Le Wang, Fang Liu

**Affiliations:** 1 Oujiang Laboratory (Zhejiang Lab for Regenerative Medicine, Vision and Brain Health), Institute of Mental Health and Drug Discovery, School of Psychiatry, Wenzhou Medical University, Wenzhou 325000, China; 2 School of Pharmaceutical Sciences, Wenzhou Medical University, Wenzhou 325000, China; 3 Campbell Family Mental Health Research Institute, Centre for Addiction and Mental Health, Toronto, ON M5T1R8, Canada; 4 Department of Pharmacology & Toxicology, Institute of Medical Science, University of Toronto, Toronto, ON M5T1R8, Canada; 5 Department of Physiology, Institute of Medical Science, University of Toronto, Toronto, ON M5T1R8, Canada; 6 Department of Psychiatry, Institute of Medical Science, University of Toronto, Toronto, ON M5T1R8, Canada

**Keywords:** ischemic stroke, mitochondrial dysfunction, NDUFB3, NDUFA4, apoptosis, JNK pathway

## Abstract

**Background:** Mitochondrial dysfunction is a central event in the pathogenesis of ischemic stroke. The roles of specific mitochondrial complex subunits, such as NDUFA4 and NDUFB3, in cerebral ischemia–reperfusion injury remain poorly defined. This study aims to investigate the dynamic expressions and functional impact of NDUFA4 and NDUFB3 in ischemic stroke. **Methods:** A transient middle cerebral artery occlusion (MCAO) model was established in male C57BL/6J mice. Label-free quantitative proteomics and Western blotting were employed to analyze protein expression in the ischemic penumbra. Highly differentiated PC12 cells were subjected to oxygen-glucose deprivation/reperfusion (OGD/R) or glutamate excitotoxicity to mimic ischemic injury in vitro. The functional consequences of NDUFB3 knockdown and overexpression were assessed by measuring ATP levels, reactive oxygen species (ROS), mitochondrial membrane potential (ΔΨm), and apoptosis. The involvement of the JNK-mediated mitochondrial apoptotic pathway was also examined. **Results:** Proteomic analysis revealed a significant upregulation of NDUFA4 and NDUFB3 in the ischemic penumbra of MCAO mice, as verified by western blot. In highly differentiated PC12 cells, both OGD/R and glutamate exposure induced a time-dependent increase in these proteins in mitochondrial fractions. Functional studies demonstrated that NDUFB3 knockdown significantly rescued OGD/R-induced mitochondrial dysfunction, as indicated by restored ATP production, reduced ROS generation, and stabilized ΔΨm. Furthermore, NDUFB3 silencing attenuated apoptosis by inhibiting JNK phosphorylation and decreasing BAX levels. Conversely, overexpression of NDUFB3 alone was sufficient to induce mitochondrial abnormalities, including loss of ΔΨm and elevated oxidative stress in highly differentiated PC12 cells. **Conclusions:** Ischemic injury triggers the upregulation of mitochondrial complex subunits NDUFA4 and NDUFB3. While this may initially act as a compensatory response, our findings identify NDUFB3 as a critical mediator of ischemic stroke pathology, whose overexpression drives mitochondrial dysfunction and apoptosis. In contrast, the suppression of NDUFB3 provides protection against ischemic injury. Therefore, NDUFB3 may be a potential candidate therapeutic target for reducing mitochondrial damage in ischemic stroke, but this role requires further validation in additional experimental and translational models.

## 1. Introduction

Ischemic stroke poses a major global public health challenge and is characterized by its high rates of disability and mortality. Emerging clinical evidence highlights that the hyper-acute phase of ischemic stroke is tightly coupled with systemic biochemical dysregulation and profound hemostatic alterations, which critically influence microvascular patency and tissue outcomes [1,2]. These systemic changes often exacerbate the localized metabolic and mitochondrial collapse within the ischemic penumbra. Nevertheless, the sudden interruption of cerebral blood flow remains the primary trigger, which depletes oxygen and glucose supply to the affected brain regions and triggers a rapid cascade of injury. Although advancements in thrombolysis and thrombectomy have successfully restored blood flow for some patients, the subsequent reperfusion injury often exacerbates neuronal death—a clinical dilemma for which no specific effective drug currently exists [3]. Within this complex pathophysiological network, mitochondrial dysfunction is widely recognized as a convergence point of the ischemic cascade and a critical “checkpoint” determining cellular fate [4,5].

Mitochondria produce ATP via oxidative phosphorylation to maintain transmembrane ion gradients and neurotransmission. Under ischemic and hypoxic conditions, the blockade of the electron transport chain (ETC) not only leads to a collapse of ATP synthesis but also triggers the loss of mitochondrial membrane potential and a surge of reactive oxygen species (ROS) release [6,7]. Among the four complexes of the ETC, Complex I is the largest and most structurally intricate enzyme complex, serving as a primary source of ROS production following ischemia. It comprises 45 subunits, including 14 core catalytic subunits and 31 accessory subunits [8,9]. Previous research has predominantly focused on the impaired enzymatic activity of the core subunits.

Interestingly, a key molecular mechanism driving ROS burst upon reperfusion is reverse electron transport (RET), defined as the upstream flow of electrons from the reduced ubiquinone pool backward into Complex I [10,11]. RET-driven ROS is a major contributor to I/R injury, and reverse electron transport at mitochondrial complex I has been established as a primary source of the reperfusion-induced ROS burst [11,12]. Moreover, brain I/R leads to a reversible loss of the flavin mononucleotide (FMN) cofactor from Complex I—an event specifically triggered by RET conditions—which further compromises enzyme activity [11]. Given that the stability of FMN binding depends on the structural integrity of Complex I, these observations raise the possibility that accessory subunits of Complex I, which help maintain structural integrity and modulate enzyme stability, may influence the susceptibility to RET-mediated damage. However, the functional roles of these accessory subunits in cerebral ischemia–reperfusion injury have remained largely unexplored.

Recent evidence suggests that these accessory subunits may regulate the stability of the entire complex under stress [13]. Two such subunits, NDUFB3 (NADH: Ubiquinone Oxidoreductase Subunit B3) and NDUFA4 (NADH Dehydrogenase 1 Alpha Subcomplex 4), have emerged as proteins of interest. NDUFB3 is a hydrophobic subunit of the Complex I membrane arm [14], while NDUFA4, historically classified with Complex I, interacts closely with Complex IV (Cytochrome c Oxidase) [15,16,17]. Although they are important for assembly and super complex formation, their specific expression dynamics and functional roles in the acute phase of cerebral ischemia–reperfusion have not been characterized. Given the central role of Complex I dysfunction in post-ischemic ROS production and energy failure, and considering that NDUFB3 is a core, hydrophobic subunit integral to Complex I assembly and stability, we prioritized investigating the pathophysiological significance of NDUFB3 upregulation in this study.

Excitotoxicity represents another hallmark of ischemic injury. Ischemia leads to the accumulation of glutamate in the synaptic cleft, thereby over-activating NMDA receptors and causing intracellular calcium overload [18]. There exists a close interaction between calcium signaling dysregulation and mitochondrial dysfunction [19]; however, it remains unclear whether excitotoxic signaling directly drives the pathological remodeling of specific mitochondrial subunits. We hypothesize that an ischemia-induced imbalance in the expression of specific mitochondrial proteins may be a key molecular event linking glutamate toxicity to the mitochondrial apoptotic pathway. This study aims to clarify the role of NDUFB3 in mitochondrial damage following ischemic stroke, providing a theoretical basis for identifying new neuroprotective targets.

## 2. Materials and Methods

### 2.1. Mice

All animal experimental procedures were approved by the Institutional Animal Care and Use Committee of Oujiang Laboratory, Zhejiang, China (OJLAB24031502) and conducted in strict compliance with the Regulations for the Administration of Affairs Concerning Experimental Animals. Sixty male C57BL/6J mice (6–8 weeks old, 20–25 g) were obtained from Zhejiang Vital River Laboratory Animal Technology Co., Ltd., Jiaxing, China. The mice were housed under controlled environmental conditions (20–23 °C) with a 12:12 h light/dark cycle and provided ad libitum access to standard rodent chow and water throughout the acclimatization and experimental periods. Animals were randomly assigned to experimental groups using a simple randomization method based on a random number table, with the allocation sequence generated prior to study initiation.

To minimize potential confounding factors, treatment order was randomized, and cage positions were randomized and rotated on a weekly basis. Investigators responsible for outcome assessment were blinded to group allocation. The individual who generated the randomization sequence was not involved in subsequent experimental procedures. In addition, both the surgeon and the statistician performing data analysis were blinded to group assignments.

### 2.2. MCAO Mouse Model

C57BL/6J mice (6–8 weeks old, 20–25 g, male) were purchased from Vital River and used to establish a transient middle cerebral artery occlusion (MCAO) model to induce focal cerebral ischemia. Mice were allowed free access to food and water for two weeks to acclimate to the laboratory environment and fasted for 12 h with water deprivation before surgery. Mice were first anesthetized with isoflurane (RWD, Shenzhen, China). After supine fixation, the neck region and surgical instruments were disinfected with povidone-iodine. A midline incision was made in the neck, and the common carotid artery, internal carotid artery, and external carotid artery were carefully dissected under a dissection microscope (Nikon, Tokyo, Japan). The external carotid artery and the proximal portion of the common carotid artery were ligated, and a slipknot was tied at the distal portion of the common carotid artery. The internal carotid artery was clamped with a microvascular clamp, and a small puncture was made between the ligated site and the slipknot using an insulin syringe needle (BD, Franklin Lakes, NJ, USA). A silicone-coated filament (RWD, China) was gently inserted into the puncture, and the slipknot was tightened. The microvascular clamp on the internal carotid artery was removed, and the filament was slowly advanced into the internal carotid artery until mild resistance was felt, occluding the right middle cerebral artery to induce cerebral ischemia. After 2 h of ischemia, the filament was withdrawn to restore blood flow, establishing an ischemia–reperfusion model. Throughout the procedure, mice were placed on a heating pad to maintain body temperature at 37 °C. To minimize postoperative pain and distress, buprenorphine (0.05 mg/kg, subcutaneously) was administered immediately after surgery and thereafter as needed. Animals were excluded from the study based on the following predefined criteria: (1) subarachnoid or intracranial hemorrhage; (2) insufficient reduction in cerebral blood flow; (3) absence of infarction. All animals were monitored at least twice daily for neurological deficits, body weight loss, and general condition. Humane endpoints were defined as >20% body weight loss, inability to access food or water, severe neurological impairment, or signs of severe distress, at which point animals were euthanized. Applying these criteria, 20–30% of animals were excluded.

### 2.3. Cell Culture and Transfection

Highly differentiated PC12 cells were cultured in complete RPMI 1640 medium and maintained in a cell incubator at 37°C with 5% CO_2_. For transfection, cells were seeded in 6-well plates and transfected with the recombinant plasmid (pcDNA3.1-CMV-NDUFB3-3×FLAG, Beijing Tsingke Biotech Co., Ltd., Beijing, China) or the empty pcDNA3.1 vector using Lipofectamine 3000 (Life Technology, Carlsbad, CA, USA) according to the manufacturer’s instructions. Cells were harvested for further analysis 48 h post-transfection. Small interfering RNA (siRNA) targeting rat *Ndufb3* (NCBI Gene ID: 301427) and a negative control siRNA were designed and synthesized by MedChemExpress, Monmouth Junction, NJ, USA. The sense and antisense sequences for rat Ndufb3 siRNA were as follows: 5′-GAAGAAGCUUGCUGCACGAGG-3′ and 5′-UCGUGCAGCAAGCUUCUUCUG-3′. Lipofectamine RNAiMAX (Life Technology) was used according to the manufacturer’s instructions. Briefly, RNA and RNAiMAX were diluted in Opti-MEM and incubated for 15 min. The transfection mix was added to the culture media. After 48 h, cells were performed to other processes.

### 2.4. Neurological Function Assessment

Neurological deficits in MCAO mice were evaluated 24 h after reperfusion using the Longa scoring scale, defined as follows: Zero points: No neurological dysfunction. One point: Contralateral forelimb adduction and flexion when lifted by the tail (mild neurological deficit). Two points: Circling toward the contralateral side during crawling (moderate neurological deficit). Three points: Falling toward the contralateral side when standing or crawling (severe neurological deficit). Four points: No spontaneous activity with altered consciousness.

### 2.5. Cerebral Blood Flow Measurement

Twenty-four hours after reperfusion, MCAO mice were anesthetized with isoflurane (RWD, China). The scalp over the entire dorsal surface of the skull was removed using forceps and surgical scissors. Cerebral blood flow (CBF) across the whole brain was measured using a laser speckle flow imaging system (RWD, Shenzhen, China). Two regions of interest (ROIs) were selected: the ipsilateral cortex (ROI 1) and the contralateral cortex (ROI 2), to quantify CBF changes. The ratio of (ROI 2 CBF − ROI 1 CBF) to ROI 2 CBF was calculated.

### 2.6. TTC Staining

Preparation of 2% TTC Staining Solution: Weigh 200 mg of TTC powder (Sigma) and dissolve it in 10 mL of PBS buffer (Solarbio, Beijing, China). Store the solution at 4 °C in the dark and prepare it freshly before use. After obtaining the brain tissue, remove the olfactory bulbs and cerebellum. Fix the tissue in the slicing chamber of a Leica vibrating microtome (Leica, Bannockburn, IL, USA) filled with PBS buffer. Perform coronal sectioning of the brain at a speed of 0.8 mm/s, cutting the brain into 5–6 slices, with each slice taken at 2 mm intervals. Immediately place the slices in the 2% TTC staining solution and incubate them at 37 °C in the dark for 30 min. Turn the slices over every 10 min to ensure uniform staining.

Observe the staining changes in the mouse brain tissue. The infarcted tissue appears white, while the surrounding normal tissue appears red. Place the stained slices in 4% paraformaldehyde (Aladdin, Shanghai, China) and store them at 4 °C in the dark for fixation.

### 2.7. Protein Extraction and Western Blot Analysis

Brain tissues were placed on a Corning^®^ culture dish (Corning Incorporated, Corning, NY, USA) lid lined with filter paper, and the target tissue (ischemic penumbra) was dissected using a blade. The samples were immediately placed on ice, and mitochondrial proteins were extracted using a Tissue Mitochondria Isolation Kit (Beyotime, Shanghai, China) according to the manufacturer’s instructions. For highly differentiated PC12 cells, after injury, cells were detached with trypsin-EDTA (0.25%, Gibco, Thermo Fisher Scientific, Waltham, MA, USA), collected, and mitochondrial proteins were extracted using a Cell Mitochondria Isolation Kit (C3601, Beyotime, Shanghai, China). Protein concentrations were determined using a BCA Protein Assay Kit (P0012, Beyotime, Shanghai, China): absorbance was measured with a microplate reader (Thermo Fisher Scientific, Waltham, MA, USA), and protein concentrations were calculated against a standard curve. Samples were mixed with 5× SDS-PAGE loading buffer (New Cell & Molecular Biotech, Suzhou, China), denatured at 95 °C for 5 min, and separated by 12.5% SDS-PAGE using a 12.5% ExpressCast PAGE Color Gel Rapid Kit (New Cell & Molecular Biotech, Suzhou, China) based on the molecular weight of the target proteins. Proteins were transferred to a PVDF membrane (Millipore, Billerica, MA, USA), blocked with 10% Difco™ Skim Milk (BD) in 1× TBST for 1 h, and incubated overnight at 4 °C with gentle shaking in primary antibodies: Anti-NDUFB3 (Santa Cruz, Dallas, TX, USA. sc-393351, 1:1000), Anti-NDUFA4 (ABclonal, Wuhan, China, A15693, 1:1000), Anti-VDAC1 (Abcam, ab15895, 1:1000), Anti- Phospho-JNK (Thr183/Tyr185) (Proteintech, Wuhan, China, 60666-1-Ig, 1:1000), Anti- tJNK (Proteintech, Wuhan, China, 51153-1-AP, 1:1000), Anti- BAX (Proteintech, Wuhan, China, 50599-2-Ig, 1:1000) and Anti-β-actin (Proteintech, Wuhan, China, 66009-1-Ig, 1:1000). Protein bands were detected using a Super-Sensitive ECL Chemiluminescence Kit (New Cell & Molecular Biotech, Suzhou, China), and grayscale values were analyzed using ImageJ software (version 1.54f).

### 2.8. Assessment of Mitochondrial Function and Cell Activity

Highly differentiated PC12 cells were seeded on poly-L-lysine-coated coverslips in 24-well plates and cultured for 24 h. Oxygen-glucose deprivation (OGD) was induced by incubating the cells in glucose-free DMEM/F12 medium (supplemented with HEPES) under hypoxic conditions (1% O_2_, 94% N_2_, 5% CO_2_) for 8 h. After 18 h of reperfusion with normoglycemic medium, intracellular reactive oxygen species (ROS) levels were measured using the Reactive Oxygen Species Assay Kit (YEASEN Biotechnology, Shanghai, China, 50101ES01), and mitochondrial membrane potential was evaluated via tetramethylrhodamine ethyl ester (TMRE) staining (Mitochondrial Membrane Potential Assay Kit, 115532-52-0, Beyotime, Shanghai, China), while cell viability was assessed by Calcein AM/PI staining (Calcein/PI Cell Viability/Cytotoxicity Assay Kit, C2015, Beyotime, Shanghai, China). Highly differentiated PC12 cells were seeded on poly-L-lysine-coated coverslips in 96-well plates and cultured for 24 h. Cells were subjected to OGD for 8 h, followed by re-oxygenation for another 18 h. ATP levels were determined using the CellTiter-Lumi™ Plus Luminescent Cell Viability Assay Kit (C0068, Beyotime, Shanghai, China) and cell viability was checked in parallel using the Cell Counting Kit-8 (HY-K0301, MedChemExpress, Shanghai, China) and Cytotoxicity LDH Assay Kit (HY-K1090, MedChemExpress, Shanghai, China). For the cell viability after NDUFB3 overexpressed, PC12 cells were seeded on 96-well plates and cultured for 24 h. After overexpression of the NDUB3 protein for 24 h, the cells were treated with 10 µM SP600125 for 12 h, followed by detection of LDH release and CCK-8 viability. All treatments were performed in at least three independent replicates.

### 2.9. Sample Collection and Preparation

The brain tissue samples were transferred into a 2 mL centrifuge tube and dissolved in lysis solution with ultrasonic. Then it was sonicated 5 min on ice using a high-intensity ultrasound processor. The supernatant was collected by centrifugation at 15,000× *g* for 15 min at 4 °C. We measured the protein concentration using BCA Kit (P0012, Beyotime Biotechnology). The 150 μg protein was obtained and dithiothreitol was added in the solution at 56 °C for 1 h. The iodoacetamide was added in the solution and stored at room temperature in dark place for 2 h. After removal of the liquid using ultrafiltration membrane (Sartorius), the sample was dissolved using 50 mM NH_4_HCO_3_. Then, trypsin (MS-grade from Sigma-Aldrich, St. Louis, MO, USA) was added at a ratio of 1: 100 (trypsin: protein), and enzymatic hydrolysis was carried out overnight at 37 °C. After that, the peptides were collected by centrifugation at 15,000× *g* for 30 min. These samples were stored at −80°C before mass spectrometry analysis. NanoDrop (Thermo Fisher Scientific, San Jose, CA, USA) was used to measure the concentration of protein digestion.

Samples were dissolved in 0.1% formic acid aqueous solution and injected for nano-LC-MS/MS analysis. The peptide mixture was loaded onto the C18-reversed phase analytical column (Thermo Fisher Scientific, Waltham, MA, USA, Acclaim Pep Map RSLC 50 µm × 15 cm, nano viper, P/N164943) in buffer A (0.1% Formic acid) and separated with a linear gradient of buffer B (80% acetonitrile and 0.1% Formic acid) at a flow rate of 400 nL/min. LC-MS/MS analysis was performed on a Orbitrap Exploris 480 mass spectrometer with the FAIMS Pro interface (Thermo Fisher Scientific, Waltham, MA, USA) that was coupled to Vanquish Core (Thermo Fisher Scientific, Waltham, MA, USA) for 90 min. The mass spectrometer was operated in positive ion mode. MS data was acquired choosing the most abundant precursor ions from the survey scan (350–2000 *m*/*z*) for HCD fragmentation. Survey scans were acquired at a resolution of 70,000 at *m*/*z* 200 and resolution for HCD spectra was set to 17,500 at *m*/*z* 200, and isolation width was 2 *m*/*z*. Normalized collision energy was 30 eV.

### 2.10. Bioinformatics Analysis

Firstly, the raw files were processed using Thermo Scientific™ Proteome Discoverer™ 3.0.1.27 software. Then, label-free quantitation (LFQ) data from the brain protein was analyzed using the SEQUEST database searches with a combination of tryptic and fixed amino acid modifications. T-test was used to analyze quantitative variables and a *p*-value < 0.05 and 2-fold changes was considered statistically significant. All functionally annotated analysis of differentially expressed proteins were enriched for analysis using the online platform of DAVID (https://davidbioinformatics.nih.gov/), and the biological information of these differential proteins was annotated and analyzed.

### 2.11. Stereotaxic Surgery and Viral Microinjection

Mice were anesthetized with isoflurane and placed in a stereotactic frame (RWD Life Science Inc., Shenzhen, China). Ophthalmic ointment was applied to prevent corneal drying during surgery. The skull was exposed, and bregma was identified as the reference point for all coordinate measurements. A high-titerlentiviral suspension (packaged in-house; Lenti-CMV-NDUFB3-EGFP or Lenti-CMV-EGFP control; titer: ≥1 × 10^8^ TU/mL) was injected into the dorsal hippocampus at a rate of 30 nL/min for a total volume of 1 μL using a 10 μL microsyringe (Gaoge, Shanghai, China). The coordinates for targeting the dorsal hippocampus: anterior–posterior (AP), −2.0 mm; medial–lateral (ML), ±1.8 mm; dorso-ventral (DV), −2.0 mm.

### 2.12. Transmission Electron Microscopy

Animals were euthanized by isoflurane, followed by intracardiac perfusion (0.9% saline). Brains were isolated and immersed in 2.5% glutaraldehyde. After fixation, gradient dehydration was performed using ethanol and propylene oxide, followed by infiltration and embedding with a mixture of acetone and resin, and 70 nm ultrathin sections were double-stained with uranyl acetate and lead citrate. Sections were imaged using transmission electron microscope (Zeiss, Oberkochen, Germany) at 30 kV. Mitochondrial morphologies were analyzed in ImageJ. At least 100 mitochondria from 7 to 8 random fields should be measured, with a primary magnification set at ×20,000. All micrographs were selected randomly to avoid bias.

### 2.13. Statistical Analysis

Data were analyzed using GraphPad Prism 9.5 software and presented as mean ± standard error of the mean (SEM). The exact number of independent biological replicates (n) for each in vitro and in vivo experiment is explicitly detailed in the respective figure legends. Prior to inferential testing, data distributions were rigorously evaluated for normality using the Shapiro–Wilk test, and homogeneity of variances was evaluated using the Brown–Forsythe test for multiple-group comparisons or the F test for two-group comparisons. For datasets meeting both assumptions, data were analyzed using Student’s unpaired t-test (two groups) or one way ANOVA (multiple groups). For normally distributed datasets with unequal variances, Welch’s *t*-test (two groups) or Welch’s ANOVA (multiple groups) was used. For datasets violating the normality assumption, the Mann–Whitney U test (two groups) or the Kruskal–Wallis test was employed. Crucially, for the high-dimensional label-free quantitative proteomic analyses, raw *p*-values were subjected to the Benjamini–Hochberg False Discovery Rate (FDR) correction to strictly mitigate false positives generated by multiple hypothesis testing, with an adjusted FDR threshold of <0.05 deemed statistically significant. Statistical significance was defined as *p* < 0.05 for all analyses. The primary outcome measure for the in vivo experiments was infarct volume assessed by TTC staining.

## 3. Results

### 3.1. Functional and Structural Validation of Middle Cerebral Artery Occlusion

**Figure 1:**
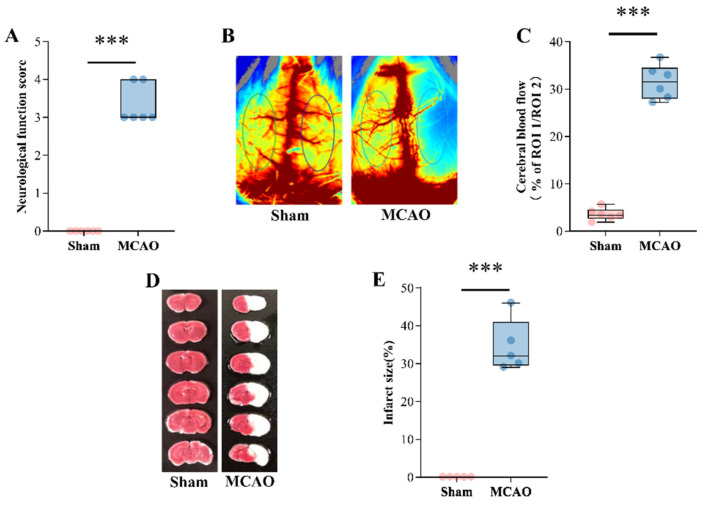
In 8-week-old C57BL/6J mice, the middle cerebral artery on the right side was occluded using a suture to establish a mouse model of ischemic stroke. After 24 h, neurological deficit scoring, cerebral blood flow measurement, and TTC staining were performed. (**A**) Longa neurological deficit scoring was carried out on each group of mice 24 h after the establishment of the MCAO mouse model (n = 6/group). (**B**) Representative images of cerebral blood flow measured by a laser speckle blood flow imaging system 24 h after the establishment of the MCAO mouse model. The regions of interest (ROI), namely the ipsilateral cortex (ROI 1) and the contralateral cortex (ROI 2), were respectively used to quantify the changes in cerebral blood flow. (**C**) The ratio of cerebral blood flow [ROI 2–ROI 1) to ROI 2] in each group (n = 6/group). (**D**) Representative slice images of TTC staining in each group 24 h after the establishment of the MCAO mouse model. The white part of the slice represents the ischemic area, and the red part represents the normal tissue. (**E**) Quantitative analysis of cerebral infarct volume in each group (n = 5/group). All data are expressed as mean ± SEM, Student’s t test, *** *p* < 0.001.

Transient focal cerebral ischemia was experimentally induced in C57BL/6J mice through 2 h right middle cerebral artery occlusion (MCAO) using an intraluminal suture, followed by a 24 h reperfusion period. Neurological deficits were assessed at 24 h post-MCAO using the Longa score. Compared with sham-operated controls, the MCAO mice exhibited significant neurological impairments (*p* = 0.0022) (Figure 1A), indicative of successful ischemic stroke modeling. Laser speckle contrast imaging demonstrated a significant reduction in cerebral blood flow within the ipsilateral hemisphere of MCAO mice versus contralateral regions (*p* < 0.001), while sham mice showed no interhemispheric perfusion differences (Figure 1B,C). Triphenyl tetrazolium chloride (TTC) staining revealed no detectable infarction in sham controls, whereas MCAO mice developed substantial ipsilateral cerebral infarcts (34.2 ± 5.8% of hemispheric volume, *p* = 0.0079 vs. sham) (Figure 1D,E). Collectively, these findings validate the MCAO model through functional, hemodynamic, and histopathological evidence of ischemic injury.

### 3.2. NDUFA4 and NDUFB3 Upregulated in the Ischemic Penumbra of MCAO Mice

**Figure 2:**
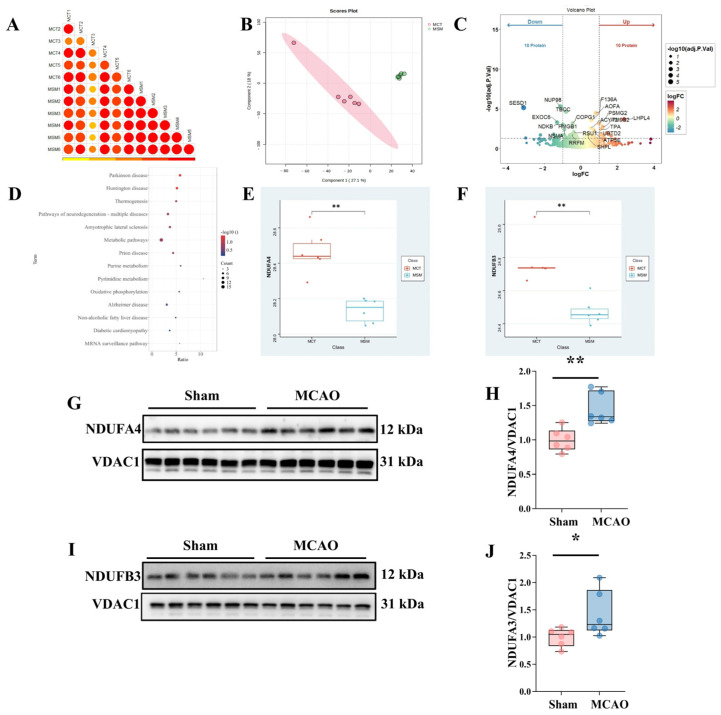
Proteomics analysis of MCAO model (MCT) and Sham (MSM) groups. (**A**) Correlation heatmap of all groups in MCAO model (MCT) and Sham (MSM) group samples; (**B**) Partial least squares-discriminate analysis (PLS-DA) scores of samples in MCAO model and Sham group samples; (**C**) MCAO model group vs. Sham group, where blue and red dots indicate down- and up-regulation in the Sham group, respectively; (**D**) The KEGG analysis of the differential proteins in the all groups; (**E**) The boxplot of NDUFA4 of MCAO model (MCT) group vs. Sham group, where the Y-axis indicates the value (peak abundance); (**F**) The boxplot of NDUFB3 of MCAO model (MCT) group vs. Sham group, where the Y-axis indicates the value (peak bun dance); (**G**) Schematic diagrams of the protein expression levels of NDUFA4 and VDAC1 in the Sham group and the MCAO group. (**H**) Quantification of NDUFA4 in the ischemic penumbra. (**I**) Schematic diagrams of the protein expression levels of NDUFB3 and VDAC1 in the Sham group and the MCAO group. (**J**) Quantification of NDUFB3 in the ischemic penumbra. The protein level was quantified relative to VDAC1 and normalized as a multiple of the Sham group. All data are expressed as mean ± SEM (n = 6/group), Student’s t test, * *p* < 0.05, ** *p* < 0.01.

Label-free quantitative proteomic analysis was performed to assess which proteins were differentially expressed after MCAO model induction. The Pearson correlation coefficients between biological repeats were all >0.92, indicating satisfactory reproducibility (Figure 2A). The PLS-DA model score plot shows clear separation in the MCAO and sham group proteomes (Figure 2B).

Following 5-fold cross-validation, the PLS-DA model built on the hippocampal proteome achieved excellent classification accuracy (R^2^Y = 0.98622; Q^2^ = 0.69969), supporting robust separation between sham and MCAO groups. Adjusted *p*-values (FDR < 0.05) were used as the threshold to limit false positives among differentially expressed proteins (Supplementary Tables S1 and S2). The MCAO model induced the downregulation of 10 and upregulation of 10 proteins compared to sham controls (Figure 2C).

We performed KEGG pathway enrichment analysis using the identified differentially expressed proteins (Figure 2D). Many neurodegenerative disease pathways were identified, including Parkinson’s disease, Huntington’s disease, and Alzheimer’s disease. To transition from broad proteomic mapping to targeted functional validation, candidate proteins were meticulously filtered based on strict dual criteria: (1) high statistical significance combined with a substantial magnitude of change (Fold Change > 2.5, Benjamini–Hochberg FDR < 0.01), and (2) their specific topological enrichment within the KEGG Oxidative Phosphorylation pathway. Consequently, the mitochondrial complex I subunits NDUFB3 and NDUFA4 emerged as the highest priority candidates, exhibiting significant upregulation in MCAO mice compared to sham controls (FC = 2.8 and 3.1, respectively; FDR < 0.01) (Figure 2E,F). This upregulation was confirmed via Western blot analysis using ischemic penumbra tissue. Compared to the sham group, NDUFA4 (*p* = 0.0031) (Figure 2G,H) and NDUFB3 (*p* = 0.0461) (Figure 2I,J) levels were higher in the MCAO group, indicating oxidative phosphorylation alterations and mitochondrial dysfunction in ischemic pathology.

### 3.3. Oxygen Glucose Deprivation/Reperfusion and Glutamate Toxicity Induced Mitochondrial Enrichment of NDUFA4 and NDUFB3 in Highly Differentiated PC12 Cells

**Figure 3:**
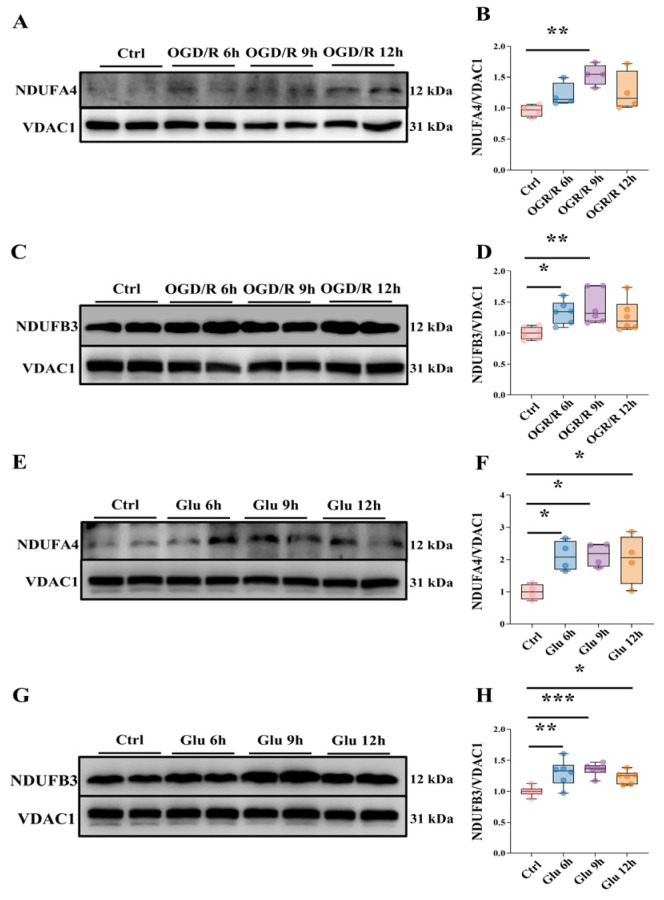
Expression Changes in NDUFA4 and NDUFB3 in highly differentiated PC12 Cells Under OGD/R and Glutamate Injury Models. (**A**) Representative blot images of mitochondrial NDUFA4 and the loading control VDAC1 in highly differentiated PC12 cells subjected to Ctrl or Oxygen-Glucose Deprivation/Reperfusion (OGD/R) for 6, 9, and 12 h. (**B**) Quantification of mitochondrial NDUFA4 protein levels normalized to VDAC1 from (**A**) (n = 4 independent experiments) (n = 4/group). (**C**) Representative blot images of mitochondrial NDUFB3 and VDAC1 under conditions identical to (**A**). (**D**) Quantification of mitochondrial NDUFB3 protein levels normalized to VDAC1 from (**C**) (n = 6/group). (**E**) Representative blot images of mitochondrial NDUFA4 and VDAC1 in highly differentiated PC12 cells treated with Glutamate (Glu) for 6, 9, and 12 h. (**F**) Quantification of mitochondrial NDUFA4 protein levels normalized to VDAC1 from (**E**) (n = 4/group). (**G**) Representative blot images of mitochondrial NDUFB3 and VDAC1 under conditions identical to (**E**). (**H**) Quantification of mitochondrial NDUFB3 protein levels normalized to VDAC1 from (**G**) (n = 6/group). All data are presented as mean ± SEM, One-way ANOVA, * *p* < 0.05, ** *p* < 0.01, *** *p* < 0.001.

To further investigate the role of NDUFB3 and NDUFA4 expression levels in ischemic injury, highly differentiated PC12 cells were exposed to Oxygen Glucose Deprivation/Reperfusion (OGD/R) or glutamate-excitotoxic injury. Western blot analysis of mitochondrial fractions showed NDUFB3 (*p* = 0.0363) and NDUFA4 expression increased slightly but not significantly after 6 h of OGD/R. At 9 h, both proteins showed a significant increase (*p* = 0.0082 and 0.0087). At 12 h, their expression started to decline (Figure 3A–D). Glutamate exposure, however, significantly increased NDUFB3 (Figure 3G,H) and NDUFA4 (Figure 3E,F) expression at all time points (6 h: *p* = 0.0029 and 0.0212, 9 h: *p* = 0.0006 and 0.0181, 12 h: *p* = 0.0217 and 0.0376). This result suggested that both ischemic and excitotoxic insults increased the mitochondrial levels of complex I subunits NDUFB3 and NDUFA4. Notably, glutamate exposure caused a more sustained upregulation, whereas OGD/R-induced expression peaked at 9 h and began to decline by 12 h, indicating distinct temporal dynamics between transient reperfusion injury and continuous excitotoxic stimulation.

### 3.4. NDUFB3 Knockdown Rescue OGD/R-Induced Mitochondrial Dysfunction in Highly Differentiated PC12 Cells

**Figure 4:**
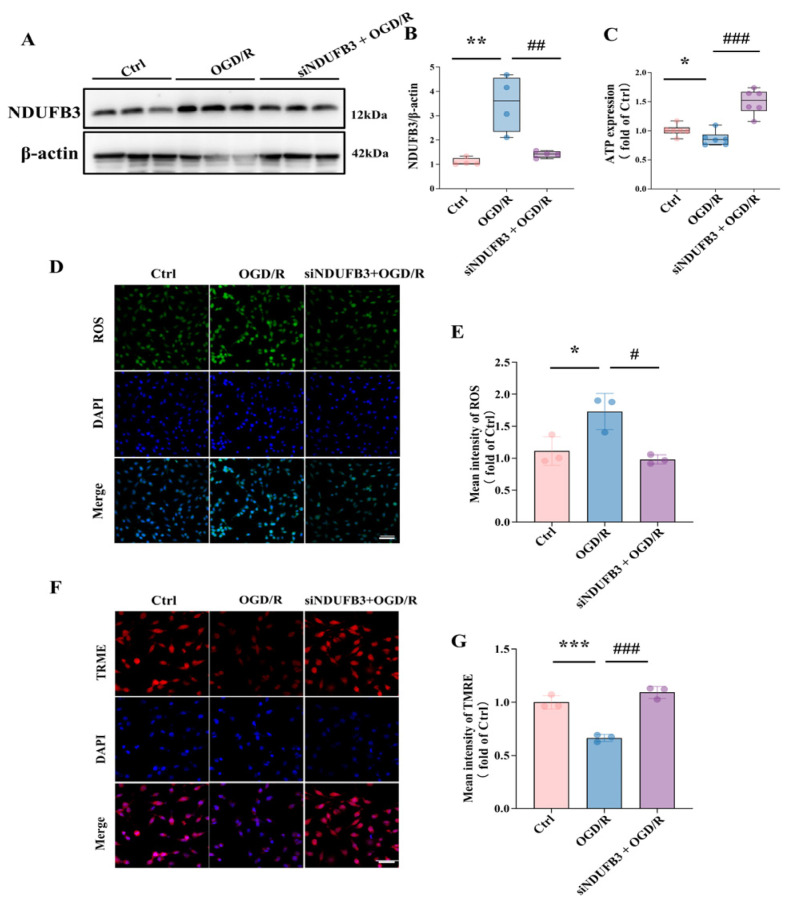
Knockdown of NDUFB3 Attenuates OGD/R-Induced Mitochondrial Dysfunction in PC12 Cells. (**A**) Representative blot images of NDUFB3 and β-actin (loading control) in PC12 cells under the following conditions: Control (Ctrl), Oxygen-Glucose Deprivation/Reperfusion (OGD/R), and OGD/R with NDUFB3 knockdown (OGD/R + siNDUFB3). (**B**) Quantitative analysis of NDUFB3 protein levels normalized to β-actin, presented as fold change relative to the Ctrl group (n = 4). (**C**) Measurement of intracellular ATP levels in the indicated groups, expressed as fold change relative to the Ctrl group (n = 5/group). (**D**) Representative immunofluorescence images of cellular reactive oxygen species (ROS, green) and nuclei (DAPI, blue) in PC12 cells. Scale bar = 50 µm. (**E**) Quantitative analysis of mean ROS fluorescence intensity from images in (**D**), expressed as fold change relative to the Ctrl group (n = 3/group, n ≥ 5 random fields per group). (**F**) Representative immunofluorescence images showing the expression of the mitochondrial TRME (red) and nuclei (DAPI, blue) in the Ctrl, OGD/R, and NDUFB3 KD groups. Scale bar = 50 µm. (**G**) Quantitative analysis of mean nuclear TRME fluorescence intensity from images in (**F**), expressed as fold change relative to the Ctrl group (n = 3/group, n ≥ 5 random fields per group). Data are presented as mean ± SEM, one-way ANOVA, * *p* < 0.05, ** *p* < 0.01, *** *p* < 0.001 vs. Ctrll; # *p* < 0.05, ## *p* < 0.01, ### *p* < 0.001 vs. OGD/R.

Next, we evaluated the effect of NDUFB3 knockdown (KD) on mitochondrial function following OGD/R in highly differentiated PC12 cells. Successful NDUFB3 KD was confirmed by Western blot analysis (Figure 4A,B). KD of NDUFB3 significantly attenuated OGD/R-induced mitochondrial dysfunction (*p* = 0.0044). Control cells subjected to OGD/R exhibited a severe depletion of ATP levels (*p* = 0.0485); however, NDUFB3 KD rescued this deficit (*p* < 0.001) (Figure 4C). Furthermore, OGD/R triggered an increase reactive oxygen species (ROS) (*p* = 0.0272), which was suppressed by NDUFB3 KD (*p* = 0.0117) (Figure 4D,E). Assessment of mitochondrial membrane potential using TMRE staining revealed NDUFB3 KD ameliorated OGD/R-induced loss of membrane integrity (*p* = 0.0001) (Figure 4F,G). These results demonstrate that reducing NDUFB3 expression protects highly differentiated PC12 cells from OGD/R-induced mitochondrial damage.

### 3.5. NDUFB3 Knockdown Attenuates OGD/R-Induced Apoptosis by Suppressing the JNK-Mediated Mitochondrial Pathway in Highly Differentiated PC12 Cells

**Figure 5:**
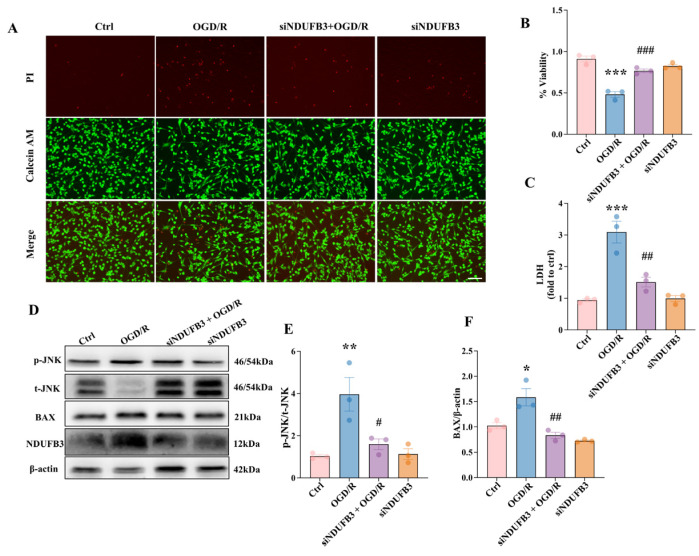
NDUFB3 Knockdown Attenuates OGD/R-Induced Neuronal Death by Suppressing the JNK-Mediated Mitochondrial Apoptotic Pathway. (**A**) Representative images of highly differentiated PC12 cells stained with Calcein AM (live cells, green) and propidium iodide (PI, dead cells, red) under the indicated conditions: Control (Ctrl), Oxygen-Glucose Deprivation/Reperfusion (OGD/R), OGD/R with NDUFB3 knockdown (siNDUFB3 + OGD/R), and siNDUFB3 alone. Scale bar = 50 µm. (**B**) Quantitative analysis of cell viability from experiments as shown in (**A**), expressed as percentage relative to the Ctrl group (n = 3/group). (**C**) Quantification of apoptotic cells by LDH assay (n = 3/group). (**D**) Representative images showing the protein levels of phosphorylated JNK (p-JNK), total JNK, BAX, and NDUFB3, with β-actin as a loading control. (**E**,**F**) Quantification of the p-JNK to total JNK ratio (**E**) and the BAX to β-actin ratio (**F**), normalized to the Ctrl group (n = 3/group). Data are presented as mean ± SEM, one-way ANOVA, * *p* < 0.05, ** *p* < 0.01, *** *p* < 0.001 vs. Ctrl; # *p* < 0.05, ## *p* < 0.01, ### *p* < 0.001 vs. OGD/R.

To determine whether inhibition of NDUFB3 expression promotes cell survival under ischemic injury, we first assessed OGD/R-induced apoptosis using Annexin V/PI staining. We found OGD/R injury significantly increased the percentage of apoptotic highly differentiated PC12 cells (*p* < 0.001). However, NDUFB3 KD suppressed apoptosis (*p* = 0.0005) (Figure 5A,B). This result was further confirmed by LDH assay (*p* = 0.0002 and 0.0020) (Figure 5C). Since OGD/R injury is known to trigger ROS overproduction and activate the JNK-mediated mitochondrial apoptotic pathway [20], we investigated the effect of NDUFB3 KD on key signaling molecules. Western blot analysis revealed that OGD/R injury increased the phosphorylation of JNK (*p* = 0.0065) (Figure 5D,E) and upregulated the pro-apoptotic protein BAX (*p* = 0.0137) (Figure 5D,F), while NDUFB3 KD reversed these changes (*p* = 0.0214 and 0.0024). These results demonstrate that NDUFB3 knockdown inhibits OGD/R-induced apoptosis in highly differentiated PC12 cells. The protective mechanism is associated with suppression of the JNK signaling pathway, likely as a downstream consequence of reduced ROS generation.

### 3.6. NDUFB3 Overexpression Induces Mitochondrial Dysfunction In Vitro and In Vivo

**Figure 6:**
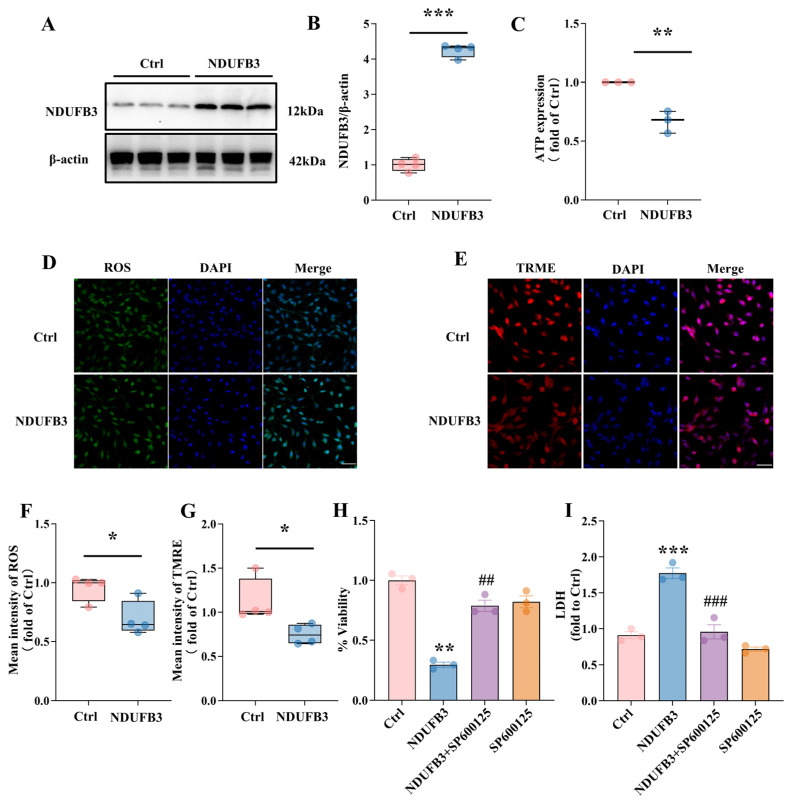
Overexpression of NDUFB3 induces mitochondrial dysfunction and promotes apoptosis via the JNK signaling pathway in highly differentiated PC12 cells. (**A**) Representative blots showing the expression of NDUFB3 and β-actin (loading control) in control (Ctrl) and NDUFB3-overexpressing (OE) highly differentiated PC12 cells (n = 4/group). (**B**) Quantification of NDUFB3 protein levels normalized to β-actin. (**C**) Measurement of intracellular ATP levels (n = 3/group). (**D**) Representative immunofluorescence images depicting cellular reactive oxygen species (ROS, green) and nuclei (DAPI, blue). Scale bar = 50 µm. (**E**) Quantification of the mean fluorescence intensity of ROS (n = 4/group). (**F**) Representative immunofluorescence images showing mitochondrial membrane potential assessed by TMRE staining (red) and nuclei (DAPI, blue). Scale bar = 50 µm. (**G**) Quantification of the mean TMRE fluorescence intensity (n = 4/group). (**H**) Cell viability and (**I**) LDH release assays in Ctrl, NDUFB3-OE, and NDUFB3-OE cells treated with the JNK inhibitor SP600125 (10 μM) (n = 3/group). Data are presented as mean ± SEM, unpaired Student’s t-test, * *p* < 0.05, ** *p* < 0.01, *** *p* < 0.001 vs. Ctrl; ## *p* < 0.01, ### *p* < 0.001 vs. NDUFB3.

To investigate the effects of elevated NDUFB3 on mitochondrial function, we established an NDUFB3-overexpressing highly differentiated PC12 cell model. Western blot analysis confirmed a significant increase in NDUFB3 protein levels compared to the control cells (*p* < 0.001) (Figure 6A,B). The overexpression of NDUFB3 was observed to cause a decline in ATP production and a significant rise in cellular ROS, as measured using MitoSOX Red staining (*p* = 0.0295) (Figure 6D,E). Furthermore, Assessment of mitochondrial membrane potential (ΔΨm) using TMRE staining [21], revealed a significant depolarization in cells overexpressing NDUFB3 compared to controls (*p* = 0.0286) (Figure 6F,G).

To determine the involvement of the JNK pathway, we treated NDUFB3-OE cells with the specific JNK inhibitor SP600125. Pharmacological inhibition of JNK significantly rescued the decreased cell viability (*p* = 0.0091) and attenuated the increased LDH release (*p* = 0.0001) caused by NDUFB3 overexpression (Figure 6H,I). Collectively, these findings demonstrate that NDUFB3 overexpression disrupts mitochondrial function and promotes apoptosis via the JNK signaling pathway.

**Figure 7:**
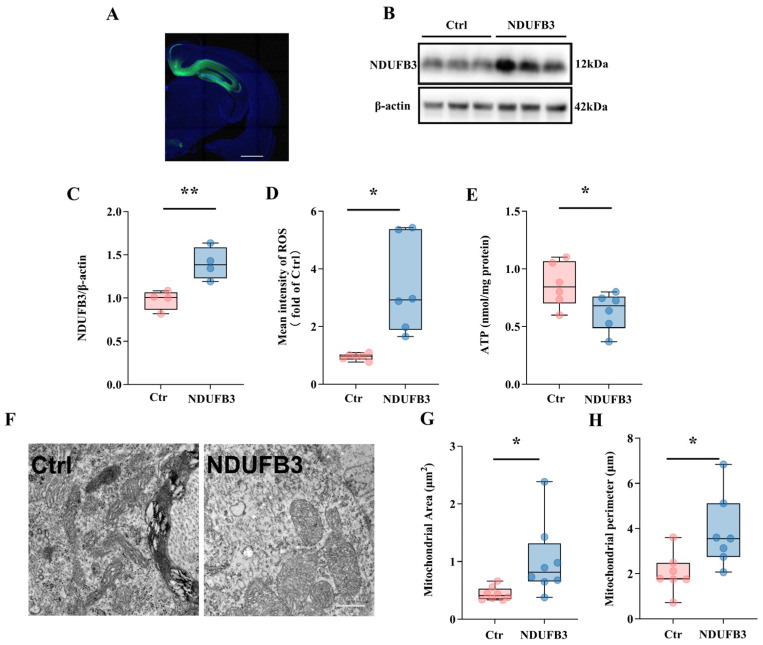
Overexpression of NDUFB3 induces mitochondrial dysfunction and structural abnormalities in the mouse brain. (**A**) Representative immunofluorescence image showing NDUFB3 expression (green) in the dorsal hippocampus of mouse. Nuclei are counterstained with DAPI (blue). Scale bar = 1000 µm. (**B**) Western blot analysis of NDUFB3 protein levels in hippocampal lysates from control (Ctrl) and NDUFB3-overexpressing (NDUFB3 OE) mice. β-actin serves as a loading control. (**C**) Quantitative analysis of the NDUFB3/β-actin protein ratio from (**B**) (n = 4/group). (**D**) Quantification of ROS levels, expressed as mean fluorescence intensity normalized to the Ctrl group (n = 6/group). (**E**) Measurement of ATP content in hippocampal tissue, normalized to total protein (n = 6/group). (**F**) Representative TEM images of hippocampal mitochondria from Ctrl (left) and NDUFB3 OE (right) mice. Scale bar = 500 nm. (**G**,**H**) Quantitative morphometric analysis of mitochondrial cross-sectional area (**G**) and mitochondrial perimeter (**H**) (n ≥ 100 mitochondria from 7 to 8 random fields). Data are presented as mean ± SEM, unpaired Student’s t-test, * *p* < 0.05, ** *p* < 0.01.

To investigate the pathophysiological role of NDUFB3 in vivo, we used lentiviral infection to overexpress NDUFB3 in the mouse hippocampal tissue (*p* = 0.0083) (Figure 7A–C). We found that NDUFB3 overexpression was associated with several mitochondrial-associated abnormalities. Oxidative stress was significantly elevated, as indicated by a significant increase in mean ROS intensity in the hippocampus from NDUFB3 overexpression (OE) mice (*p* = 0.0152) (Figure 7D). ATP levels were significantly depleted in the NDUFB3 OE group (*p* = 0.0493) (Figure 7E). Transmission electron microscopy (TEM) revealed mitochondria from NDUFB3 OE mice displayed pronounced swelling and disruption of the internal cristae structure (Figure 7F). This observation was confirmed by quantitative morphometry, which demonstrated a significant increase in both mitochondrial area and perimeter in the OE group (*p* = 0.0373 and 0.0215) (Figure 7G,H). These data demonstrate that overexpression of NDUFB3 can cause mitochondrial damage in vivo, characterized by structural deformation, bioenergetic failure, and increased oxidative stress.

## 4. Discussion

In the present study, by combining in vivo proteomic screening with in vitro mechanistic validation, we reveal that pathological upregulation of the mitochondrial Complex I subunit NDUFB3 is a critical driver of ischemia-induced mitochondrial dysfunction and apoptosis. Our findings primarily elucidate three layers of scientific inquiry: the specific remodeling of the mitochondrial proteome following ischemia, the regulatory mechanisms by which excitotoxicity affects mitochondrial subunits, and the NDUFB3-mediated ROS-JNK apoptotic cascade.

### 4.1. Pathological Upregulation of NDUFB3 and NDUFA4 in the Ischemic Penumbra

Prevailing views on ischemic injury postulate that metabolic stress predominantly triggers mitochondrial swelling, fragmentation, and widespread protein degradation [22,23]. However, our label-free quantitative proteomics challenged this conventional “damage-induced loss” paradigm, revealing a significant upregulation of NDUFB3 and NDUFA4 in the ischemic penumbra of MCAO mice 24 h post-ischemia. KEGG enrichment analyses indicated that these differentially expressed proteins are primarily clustered in pathways related to neurodegenerative diseases (e.g., Parkinson’s and Alzheimer’s disease) and oxidative phosphorylation, suggesting a shared mechanistic etiology involving metabolic failure between acute ischemic injury and chronic neurodegeneration. While both NDUFB3 (a core hydrophobic subunit of Complex I) and NDUFA4 (recently reclassified as a subunit of Complex IV) were significantly upregulated, the present study focused its subsequent mechanistic investigation on NDUFB3. This focus was driven by our central interest in Complex I dysfunction as a key early event in ischemic injury, the established role of NDUFB3 in Complex I integrity, and preliminary data indicates its potent effects on mitochondrial physiology. The specific role of NDUFA4 upregulation in the context of Complex IV function and stroke pathology warrants separate and detailed investigation.

Based on the observed temporal expression pattern and its detrimental functional consequences, we hypothesize that this acute upregulation may initially represent a maladaptive compensatory response. In the initial phase of ischemia, neurons sense ATP depletion and attempt to restore energy supply by upregulating ETC components. However, in an environment characterized by hypoxia and impaired assembly factors, the overproduced NDUFB3 cannot be correctly incorporated into Complex I. Instead, it accumulates as unstable free subunits. Given that NDUFB3 is a hydrophobic transmembrane subunit, its aberrant accumulation likely disrupts the stability of the lipid bilayer [24], a mechanism consistent with the mitochondrial membrane depolarization observed in our study. This proposed sequence of events, however, requires direct experimental validation in future work.

It is worth noting that while NDUFB3 expression remained persistently elevated under continuous glutamate toxicity, it exhibited a decline at 12 h of reperfusion in the OGD/R model. We interpret this decline not as a protective response of the cell, but rather as a hallmark of late-stage mitochondrial collapse and metabolic exhaustion. The pathogenic window of NDUFB3 upregulation primarily operates during the 6–9 h period, during which it drives the ROS-JNK-BAX cascade past the point of no return. By 12 h, when the apoptotic program is fully executed and mitochondria exhibit severe swelling and cristae disruption (as observed in our TEM data), the cellular translation machinery faces shutdown, and damaged mitochondrial components undergo non-specific degradation or mitophagy [25]. Conversely, the sustained high level of NDUFB3 in the glutamate model is likely attributed to persistent excitotoxicity and calcium overload [19,26], which continuously hyper-activates biogenesis-related transcription factors without the phase-shifting recovery window typical of reperfusion models. This temporal divergence highlights that while the initiation of NDUFB3-mediated damage is rapid, its subsequent expression profile depends heavily on the persistence of the upstream pathological stimuli.

### 4.2. Excitotoxicity as the Upstream Driver of NDUFB3 Upregulation

Another significant finding of this study is the direct link between glutamate excitotoxicity and mitochondrial subunit remodeling. Extensive preclinical evidence identifies NMDAR overactivation as the initiating factor in post-ischemic neuronal death [27]. Our in vitro data demonstrated that glutamate treatment alone was sufficient to recapitulate the time-dependent upregulation of NDUFB3 and NDUFA4 in highly differentiated PC12 cells (Figure 3). This indicates that the rise in NDUFB3 is not merely a passive consequence of hypoxia but a downstream event of the amplifying excitatory neurotransmitter signaling cascade. Calcium overload is the probable mediator of this process [28]. It is well-documented that the intracellular calcium surge triggered by glutamate receptor activation can regulate mitochondrial biogenesis-related transcription factors (e.g., PGC-1α or c-Myc) [29,30]. However, under pathological conditions, this regulation becomes dysregulated, leading to the toxic accumulation of NDUFB3. This finding tightly couples cell surface receptor events (excitotoxicity) with intracellular organelle proteostasis (mitochondrial dysfunction). While the differentiated PC12 cell line is widely used for mechanistic studies, it does not fully recapitulate the morphological, electrophysiological, or synaptic complexity of primary neurons. Furthermore, it lacks the multicellular environment (including astrocytes, microglia, and endothelial cells) that actively contributes to the pathophysiology of cerebral ischemia–reperfusion injury. Therefore, our in vitro findings should be interpreted with caution.

### 4.3. NDUFB3 Executes the Apoptotic Program via the ROS-JNK Axis

Distinguishing between an “epiphenomenon” and a “causative factor” is crucial in mechanistic research. Through gain-of-function (OE) and loss-of-function (KD) experiments, we established the core pathogenic role of NDUFB3 in ischemic injury. OE of NDUFB3 was sufficient to induce mitochondrial depolarization and ROS bursts in normal cells; conversely, NDUFB3 knockdown significantly reversed OGD/R-induced ATP depletion and apoptosis. Although the in vivo overexpression model recapitulates this ischemia-induced elevation and demonstrates that NDUFB3 alone is sufficient to elicit mitochondrial abnormalities and neuronal damage, it does not recapitulate the full complexity of cerebral I/R injury. Rather, it models the pro-apoptotic and pro-oxidative consequences of pathological NDUFB3 upregulation, which would likely be amplified under true I/R conditions by converging stressors such as energy failure and calcium overload. Accordingly, NDUFB3 overexpression should be interpreted as a specific molecular insult that mirrors a key pathogenic factor of stroke rather than as a complete recapitulation of the entire I/R cascade. Further, mechanistic exploration placed NDUFB3 upstream of the JNK-mediated mitochondrial apoptotic pathway.

ROS is a potent activator of JNK phosphorylation [31,32]. Under ischemic conditions, the aberrant accumulation of NDUFB3 leads to electron leakage from the ETC, generating excessive ROS, which subsequently activates the JNK pathway [33]. Phosphorylated JNK translocates to the mitochondria, where it inactivates BCL-2 via phosphorylation and promotes BAX oligomerization on the outer mitochondrial membrane, ultimately leading to mitochondrial outer membrane permeabilization (MOMP) and the release of pro-apoptotic factors [34,35]. Our data show that silencing NDUFB3 effectively breaks this cycle: it attenuates ROS levels, inhibits JNK phosphorylation, thereby conferring neuronal resistance to ischemic insult. Further, pharmacological inhibition of JNK with SP600125 rescued NDUFB3-OE-induced cell death (Figure 6H,I), providing direct evidence that JNK activation is required downstream of NDUFB3. Collectively, these gain-of-function, loss-of-function, and pharmacological inhibition experiments establish that NDUFB3 may act upstream of the JNK-mediated mitochondrial apoptotic pathway.

Our finding that NDUFB3 overexpression triggers ROS bursts and mitochondrial depolarization aligns with the established RET-FMN loss mechanism [10,11]. Accessory subunits such as NDUFB3 are essential for maintaining the structural integrity of Complex I [13]. Since FMN binding is sensitive to conformational changes in the enzyme [11], pathological upregulation of NDUFB3 may destabilize the FMN-binding pocket, sensitizing Complex I to RET-induced FMN loss and amplifying ROS production upon reperfusion. This positions NDUFB3 as a previously unrecognized modulator of the RET-ROS axis in ischemic stroke.

## 5. Conclusions

In summary, using MCAO and OGD/R models, this study identifies a novel mechanism in which ischemic stroke induces pathological upregulation of the mitochondrial Complex I subunit NDUFB3. The overexpression of NDUFB3 exacerbates neuronal injury by triggering ROS bursts and activating the JNK-mitochondrial apoptosis pathway. Consequently, NDUFB3 emerges not only as a potential biomarker of ischemic brain injury but also as a candidate therapeutic target for mitigating mitochondrial dysfunction and blocking reperfusion injury. But there are necessary to validate its efficacy and therapeutic window in more complex experimental and translational models of stroke before clinical consideration. Future studies should further investigate the specific transcription factors and E3 ubiquitin ligases governing NDUFB3 transcription and degradation to develop more precise molecular intervention strategies.

## Data Availability

The original contributions presented in this study are included in the article/Supplementary Materials. Further inquiries can be directed to the corresponding authors.

## Supplementary Materials

The following supporting information can be downloaded at: https://www.mdpi.com/article/10.3390/cells15121071/s1, Table S1: MCAO-sham ipsilateral Differential Proteins Expression; Table S2: MCAO-sham contralateral Differential Proteins Expression.

## Abbreviations

The following abbreviations are used in this manuscript:

MCAO	right middle cerebral artery occlusion;
OGD/R	Oxygen-glucose deprivation/reperfusion
NDUFB3	NADH:Ubiquinone Oxidoreductase Subunit B3
NDUFA4	NADH Dehydrogenase 1 Alpha Subcomplex 4
TTC	Triphenyl tetrazolium chloride
RO	Reactive oxygen species
TMRE	Tetramethylrhodamine ethyl ester
